# Layer-by-layer pH-sensitive nanoparticles for drug delivery and controlled release with improved therapeutic efficacy *in vivo*

**DOI:** 10.1080/10717544.2019.1709922

**Published:** 2020-01-10

**Authors:** Wanfu Men, Peiyao Zhu, Siyuan Dong, Wenke Liu, Kun Zhou, Yu Bai, Xiangli Liu, Shulei Gong, Shuguang Zhang

**Affiliations:** Department of Thoracic Surgery, The First Affiliated Hospital of China Medical University, Shenyang, People's Republic of China

**Keywords:** Drug delivery, layer-by-layer, nanoparticle, pH-sensitivity, HA-targeting, controlled release, cancer therapy

## Abstract

In this work, a pH-sensitive liposome–polymer nanoparticle (NP) composed of lipid, hyaluronic acid (HA) and poly(β-amino ester) (PBAE) was prepared using layer-by-layer (LbL) method for doxorubicin (DOX) targeted delivery and controlled release to enhance the cancer treatment efficacy. The NP with pH-sensitivity and targeting effect was successfully prepared by validation of charge reversal and increase of hydrodynamic diameter after each deposition of functional layer. We further showed the DOX-loaded NP had higher drug loading capacity, suitable particle size, spherical morphology, good uniformity, and high serum stability for drug delivery. We confirmed that the drug release profile was triggered by low pH with sustained release manner *in vitro*. Confocal microscopy research demonstrated that the NP was able to effectively target and deliver DOX into human non-small cell lung carcinoma (A549) cells in comparison to free DOX. Moreover, the blank NP showed negligible cytotoxicity, and the DOX-loaded NP could efficiently induce the apoptosis of A549 cells as well as free DOX. Notably, *in vivo* experiment results showed that the DOX-loaded NPs effectively inhibited the growth of tumor, enhanced the survival of tumor-bearing mice and improved the therapeutic efficacy with reduced side-effect comparing with free drug. Therefore, the NP could be a potential intelligent anticancer drug delivery carrier for cancer chemotherapy, and the LbL method might be a useful strategy to prepare multi-functional platform for drug delivery.

## 1. Introduction

Chemotherapy is still the most effective and efficient way to treat cancer in clinic even with the rapid development of nanotechnology recently (Galluzzi et al., 2015; Hallaj-Nezhadi & Hassan, 2015; Gandhi et al., 2018; Srinivasan et al., 2018). A series of chemical anticancer drugs have been well developed and clinically used in these decades, such as doxorubicin (DOX) (Zhang et al., 2012; Fabbri et al., 2016), paclitaxel (PTX) (Markman & Mekhail, 2002; Yang et al., 2018), and camptothecin (CPT) (Venditto & Simanek, 2010; Llinàs et al., 2018); however, these drugs are limited in the further clinical applications due to the serious side-effects caused by off-targeting and low therapeutic efficacy (Jungk et al., 2016; Yoshizawa et al., 2016). To overcome these obstacles, nanoscale drug delivery systems (DDSs) have attracted more and more attention and been extensively investigated (Chen et al., 2014), such as polymeric micelles (PMs), nanoparticles (NPs), prodrug, and liposome (Zhang et al., 2016; Zylberberg & Matosevic, 2016; Huang et al., 2018; Li et al., 2018; Dong et al., 2019). These effective DDSs are used to deliver hydrophobic or hydrophilic therapeutics which exhibit poor pharmacokinetics and high cytotoxicity to the site of tumor (Wang et al., 2016; Qin et al., 2017; Li et al., 2019). Recently, multilayered liposome–polymer NPs prepared by layer-by-layer (LbL) deposition technique appear as the more promising nano-sized carriers for targeted drug delivery and controlled release (Ariga et al., 2014; Borges & Mano, 2014; Sakr et al., 2016; Olszyna et al., 2019). LbL NPs are constituted by a functional core for drug loading, a multifunctional polyelectrolyte multilayer for drug controlled release, and a stealth layer for extended circulation time and targeting effect (Yan et al., 2011; Alotaibi et al., 2019). For example, Deng et al. prepared a multifunctional NP composed of liposome, polylactic acid (PLA) or polyetherimide (PEI) and hyaluronic acid (HA) via LbL technique for co-delivery of DOX and siRNA to treat the potential triple-negative breast cancer (Deng et al., 2013). Xie et al. modified the bovine serum albumin (BSA) NPs with poly(allylamine hydrochloride) (PAH)/sodium poly(4-styrene sulfonate) (PSS) multi-layers and aptamers to improve the stability and targeting ability of drug-loaded NPs (Xie et al., 2012).

Tumor cells exhibit lots of characteristic features due to the disordered metabolic profile compared to the normal cells (Mura et al., 2013), such as weak acidity, high specific enzyme and over-expressed proteins (Wang et al., 2003; Zhang et al., 2018; Raza et al., 2019). For example, the poor oxygen perfusion in most kinds of tumor cells causes the elevated levels of lactic acid, resulting in weakly acidic extracellular and intracellular tumor microenvironments (Wojtkowiak et al., 2011). Lots of researchers have reported that the weakly acidic microenvironment could be used as the specific cue for anticancer drug delivery and controlled release (Huang et al., 2017; Niu et al., 2018; Farjadian et al., 2019; Limeres et al., 2019). For instance, Zhang et al. reported a pH-sensitive PM which was self-assembled from poly(ethylene glycol) methyl ether-*b*-peptide-*g*-cholesterol (mPEG-*b*-P-*g*-Chol) for DOX delivery and controlled release. The DOX-loaded PMs were able to accumulate at the site of tumor due to the enhanced permeability and retention (EPR) effect and respond to the specific low pH for drug release (Zhang et al., 2016). Qiu group developed a cationic complex by the combination of ultrasound-targeted microbubble destruction (UTMD) with polyethylenimine (PEI) which could enhance gene transfection *in vivo*, and illuminate the effects of gene silencing. The complexes could accumulate at the site of tumor and effectively inhibit the growth of tumor (Chen et al., 2010). Meanwhile, many kinds of tumor cells, such as breast cancer stem cells (BCSCs) and human non-small cell lung carcinoma (A549), over-expressed CD44 cell surface marker (Pham et al., 2011; Ganesh et al., 2013). As reported, CD44 in the basal layer of tumor cellular epidermis could be specifically recognized by HA (Misra et al., 2015), indicating that the over-expressed CD44 on the surface of tumor cells could be used as a specific receptor for targeted drug delivery. For example, Hu et al. prepared a HA modified DOX-loaded NP based on an amphiphilic copolymer hyaluronic acid-cystamine-polylactic-co-glycolic acid (HA-ss-PLGA) which was able to actively target to the BCSCs for cancer chemotherapy. The results demonstrated that the HA-modified NPs could significantly enhance the therapeutic efficacy comparing with the negative control (Hu et al., 2015). Urbiola et al. developed a novel HA-polyamidoamine (PAMAM) system (P-HA) to enhance gene transfection in overexpressing CD44-receptor cancer cells, thereby improving the tumor therapeutic efficacy (Urbiola et al., 2014).

**Figure 1.**
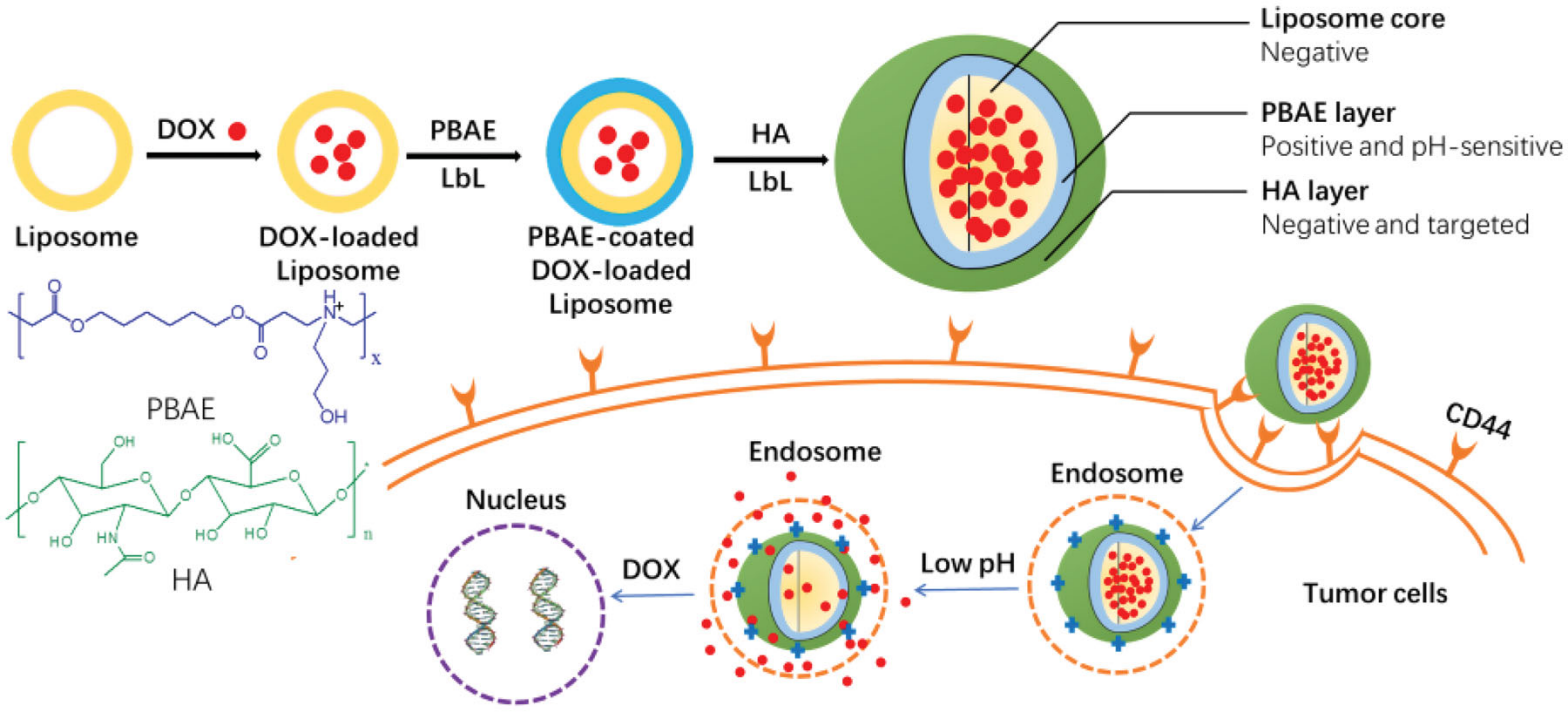
Schematic illustration of preparation process of LbL DOX-loaded NPs and targeted drug delivery for anticancer.

In this study, inspired by the specific cues in tumor microenvironment and overexpressing CD44-receptor on the surface of tumor cells, we designed and prepared the liposome–polymer NPs through LbL technique for chemical anticancer small molecule drug targeted delivery and controlled release for cancer chemotherapy (Figure 1). Here, broad-spectrum anticancer drug DOX was used as the model drug, and loaded into the liposome core which was prepared from lipids. The polymer poly(β-amino ester) (PBAE) which has been widely used in drug and/or gene targeted delivery for cancer therapy was selected as pH-responsive layer for drug controlled release (Zhang et al., 2014; Riera et al., 2019). HA, a main component in the extracellular matrix of connective tissue, was selected to form the outside shell of NPs (Lee et al., 2013). The resulted DOX-loaded NPs were able to escape the clearance by reticuloendothelial systems (RESs), exhibit prolonged circulation time, target to the site of tumor and respond to the low pH for drug delivery and controlled release (Figure 1). The cytotoxicity of NPs should be very low, and the toxic effect of DOX-loaded NPs should be close to of free DOX against the tumor cells. The DOX-loaded NPs could effectively inhibit the growth of tumor *in vivo*. Furthermore, the physicochemical properties of NPs, such as zeta-potential, hydrodynamic diameter, drug loading capacity, serum stability, and pH-triggered drug release profiles, etc., would be evaluated and investigated here.

## 2. Materials and methods

### 2.1. Materials

Hexane-1,6-dioldiacrylate (HDD, 99%), 3-amino-1-propanol (AP, 99%), HA, dimethyl sulfoxide (DMSO), dichloromethane (DCM), tetrahydrofuran (THF), and chloroform were purchased from Sigma Chemical Co. (St. Louis, MO). Doxorubicin hydrochloride (DOX-HCl) was purchased from Wuhan Yuan Cheng Gong Chuang Co. Ltd (Wuhan, China). 1,2-Distearoyl-*sn*-glycero-3-phosphocholine (DSPC) and 1-palmitoyl-2-oleoyl-phosphatidylglycerol (POPG) and cholesterol were purchased from Avanti Polar Lipids (Alabaster, AL). Methylthiazoltetrazolium (MTT) was purchased from Sigma-Aldrich (St. Louis, MO). Dulbecco’s modified Eagle’s medium (DMEM) growth media, fetal bovine serum (FBS), trypsin, penicillin, and streptomycin, were all purchased from Invitrogen (Carlsbad, CA). Human non-small cell lung carcinoma A549 cell lines were obtained from the American Type Culture Collection (ATCC). All other chemical and biological reagents were used as received.

### 2.2. Synthesis of PBAE

The cationic polymer PBAE was synthesized via Michael-type polymerization according to the previous reference (Little et al., 2005). The chemical structure and molecule weight of PBAE were confirmed using proton nuclear magnetic resonance (^1^H NMR) and gel permeation chromatography (GPC).

### 2.3. Preparation of DOX-loaded liposome

The DOX-loaded liposomes were prepared according to previously reported with few modifications (Poon et al., 2011). Briefly, three kinds of lipids cholesterol, DSPC, and POPG and at a mass ratio of 2:6:2 were dissolved into mixed solvents (chloroform:methanol = 2:1, v/v) with gently vortex in round-bottom flask. Then, a thin organic film was prepared by rotary evaporation at 60 °C for 45 min. After complete removal of chloroform, the lipid film was hydrated at 65–75 °C in citric acid buffer (pH 5.0) for 60 min under sonication. After filtration using 0.2 μm PES syringe filter, sodium carbonate (NaCO_3_) buffer was used to adjust the pH of liposomal suspension to about 6.5. For DOX loading, DOX (1 mg per 20 mg liposome) was added in a 0.9% sodium chloride solution to load via a pH gradient method as reported (Li et al., 1998; Sanson et al., 2010). The final DOX-loaded liposomes were received after purification through centrifugal filtration (100 K MWCO Millipore, Billerica, MA) three times. The feed ratio of DOX was changed to 1 mg per 15 mg and 1 mg per 10 mg, and the purification was repeated as aforementioned. The DOX-loaded liposomes were stored at −20 °C for further experiments.

### 2.4. Preparation of DOX-loaded NPs

The DOX-loaded NPs were prepared as reported (Poon et al., 2011; Deshmukh et al., 2013; Morton et al., 2013; Ramasamy et al., 2014). Briefly, 1 mL DOX-loaded liposomes (2 mg/mL) were mixed with PBAE (6 mg) in phosphate buffer solution (PBS). The mixed solution was facilitated by bath sonication (3–5 s). Then, the solution was purified through centrifugation at 2000×*g* for 20–30 min. The HA (6 mg) layer was coated on the surface of NPs similarly. The particle size and zeta-potential of each layer were recorded to validate the coating of each layer.

### 2.5. Characterization

Proton nuclear magnetic resonance (^1^H NMR) spectra measurements were operated on a spectrometer (AVANCE III 400, Bruker, 250 MHz, Fällanden, Switzerland) at 25 °C. The deuterated chloroform (CDCl_3_-*d*) with tetramethylsilane (TMS) was used as solvent.

The number average molecular weight (*M_n_*) of polymer was measured by GPC operating on an Agilent 1200 series GPC system (Palo Alto, CA) and RI detector with THF as mobile phase at a flow rate of 1.0 mL/min.

The morphology of the particles was determined by transmission electron microscopy (TEM, Hitachi H-7650, Tokyo, Japan).

### 2.6. Drug loading capacity

The drug loading content (LC) and encapsulated efficiency (EE) were measured by UV-vis spectrophotometer (UV-2450, Shimadzu, Kyoto, Japan) at 480 nm. Briefly, 0.5 mL of DOX-loaded NPs (2 mg/mL) was added into 10 mL of DMSO with gently stirring. The solution was incubated at room temperature for 1 h. The sample was recorded by UV–vis, and the concentration of DOX was confirmed according to the standard curve. The LC was defined as the weight ratio of loaded DOX to the LbL DOX-loaded NPs. The EE was defined as the weight ratio of loaded DOX to DOX in feed.

### 2.7. DLS measurement

The hydrodynamic diameter and zeta-potential of DOX-loaded liposomes, PBAE coated DOX-loaded NPs, and HA/PBAE coated DOX-loaded NPs were measured by dynamic light scattering (DLS, Malvern Zetasizer Nano S, Malvern, UK). Briefly, 50 μL of NPs was re-suspended into 1 mL of deionized water, and the samples were measured in a 1.0 mL quartz cuvette using a diode laser of 670 nm at room temperature with the scattering angle 90°.

To evaluate the serum stability of DOX-loaded NPs, 1 mL of DOX-loaded NPs (2 mg/mL) was re-suspended into 1 mL of PBS with 20% FBS. After incubation at 37 °C for different time, the particle size and polydispersity index (PDI) of sample were measured as aforementioned.

To evaluate the pH-sensitivity of DOX-loaded NPs, 1 mL of DOX-loaded NPs (2 mg/mL) was re-suspended into 1 mL of PBS at different pH values. After incubation at 37 °C for 2 h, the particle size, PDI, and zeta-potential of sample were measured as aforementioned.

### 2.8. Potentiometric titration

To measure the base dissociation constant (p*K*_b_) of polymer PBAE, potentiometric titration was operated as reported (Fernando et al., 2018). Briefly, the PBAE was dissolved in deionized water at pH 3.0. Then, sodium hydroxide (NaOH) solution was added dropwise into the mixed solution, and the real-time pH values were recorded by an automatic titration titrator (Hanon T-860, Jinan, China). The p*K*_b_ value of PBAE was determined according to the plots of pH value against the volume of NaOH solution.

### 2.9. *In vitro* release of DOX from DOX-loaded NPs

The *in vitro* release of DOX from DOX-loaded NPs was operated using dialysis method. In brief, 2 mL DOX-loaded NPs (2 mg/mL) was dissolved into 4 mL in PBS at pH 7.4 or 5.0, and the solution was transferred into a cellulose dialysis bag (MWCO 3500–4000), followed by immersing into the corresponding buffer (46 mL) in a beaker. The experiment was carried out at 37 °C with stirring 110 rpm. At pre-determined time-point, 1 mL of solution was taken for UV–vis spectrophotometry measurement, and 1 mL of fresh PBS was added. The cumulative drug release percent (*E*_r_) was calculated to the following equation:

$$E_{r}(\%)=\frac{V_{e}\sum_{1}^{n-1}C_{i}+V_{0}C_{n}}{m_{\text{DOX}}}\times 100\%$$

where *m*_DOX_ is the weight of loaded DOX, *V*_e_ is the volume of buffer in dialysis bag (4 mL), *V*_0_ is the total volume of buffer in the beaker (50 mL), and *C_i_* is the DOX concentration in the *i*th sample.

### 2.10. Cell culture

The A549 cells were cultured in fresh DMEM medium supplemented with 10% (v/v) FBS, 100 units/mL penicillin, and 100 µg/mL streptomycin. The cells were incubated at 37 °C in a CO_2_ (5%) incubator. The cells were allowed to grow until confluence and were trypsinized and seeded in plates for further experiment.

### 2.11. Confocal microscopy study

The cellular uptake and intracellular distribution of free DOX and DOX-loaded NPs in A549 cells were confirmed by confocal laser scanning microscopy (CLSM). In brief, A549 cells were grown on 60 φ culture dishes (1 × 10^5^ cells/well) in DMEM and cultured overnight. After that, the medium was replaced with fresh one. The cells were treated with free DOX or DOX-loaded NPs (10 μg/mL of DOX). After incubation for 1 h or 8 h, the dishes were washed with cold PBS three times and fixed with 4% formaldehyde for 30 min at 4 °C. The cells were incubated with DAPI after washing with PBS solution for three times. The sample was monitored by confocal laser scanning microscopy (CLSM, Zeiss, LSM 510, Oberkochen, Germany).

### 2.12. Cytotoxicity test

The cytotoxicity of liposome, NPs, free DOX, and DOX-loaded NPs against A549 cells was evaluated by standard MTT assay (Yuan et al., 2008; Dev et al., 2010). Briefly, A549 cells were seeded into a 96-well plate at an initial density of 5 × 10^3^ cells/well in DMEM and cultured in incubator overnight. The medium was removed, and a series of doses of liposomes, NPs, free DOX, and DOX-loaded NPs (200 µL/well) was added. The 96-well plate was cultured in incubator for 24 h. After addition of 20 µL of MTT solution, the plate was shaken for 5 min at 150 rpm, and then cultured for extra 4 h in incubator. After removal of the medium, 200 µL of DMSO was added in each well. The plate was gently agitated for 15 min, and the absorbance of sample was measured at 570 nm and 630 nm by a microplate reader (FL600, Bio-Tek Inc., Winooski, VT). The cell viability (%) was defined as the absorbance ratio of difference between sample and blank and difference between control and blank.

### 2.13. Therapeutic efficiency experiment

In order to evaluate the therapeutic efficacy of DOX-loaded NPs, female BALB/c-nu nude mice (5–6 weeks, Beijing Vitalriver Experimental Animal Technology Co. Ltd., Beijing, China) were used as hosts for tumor xenografts. The animal study procedures were approved by the Institutional Animal Care and Use Committee (IACUC) at China Medical University and carried out under legal protocols. A549 cells (1 × 10^6^) were subcutaneously inoculated in the left leg of each mouse. When the tumor volume reached approximately 100 mm^3^, the mice were randomly divided into three groups (PBS-, free DOX and DOX-loaded NPs-treatment, i.v. administration, *n* = 10). The mice were injected via the tail vein at DOX dose of 4 mg/kg. The tumor volume, the body weight, and survival were recorded. The tumor volume was measured by Vernier calipers and defined as (the square of width times length)/2.

### 2.14. Blood biochemistry

After different treatments, the major organs of mice were harvested carefully and weighted. The blood was collected, and separated by centrifugation with 800×*g* into cellular and plasma fractions for blood biochemical analysis.

### 2.15. Statistical analysis

All data were expressed as the mean ± standard deviation (SD). Statistical analysis was conducted using paired Students’s *t*-test or ANOVA analyses, and considered to be significant when the *p*< .05.

## 3. Results and discussion

### 3.1. Preparation and characterization of NPs and DOX-loaded NPs

To prepare the designed multifunctional NPs, the pH-sensitive polymer PBAE was first synthesized using the Michael-type polymerization. As shown in Figure S1, HDD and AP were used as diacrylate and diamine, respectively. After polymerization, the PBAE was received. The chemical structure and molecule weight of PBAE were confirmed using ^1^H NMR and GPC, respectively, as shown in Figures S2 and S3. In Figure S2, the signals at 4.05 ppm (a) were ascribed to the protons in O=C–O–C*H*_2_– in the main chain of PBAE. The signals at 3.71 ppm (h) were attributed to the vibration of protons in –C*H*_2_–OH. The peaks of 2.81 ppm (e), 2.72 ppm (f), and 2.68 ppm (d) were caused by the protons in O=C–CH_2_–C*H*_2_–N, HO–CH_2_–CH_2_–C*H*_2_–N, and O=C–C*H*_2_–CH_2_–N, respectively. The signals at 1.5–1.75 ppm (b and g) were ascribed to the protons in O=C–O–CH_2_–C*H*_2_– and HO–CH_2_–C*H*_2_–CH_2_–N. The signal at 1.41 ppm (c) was the characteristic peak of protons in O=C–O–CH_2_–CH_2_–C*H*_2_–. Next, we determined the number average molecule weight (*M_n_*) of PBAE using GPC method. The result is shown in Figure S3, and the *M_n_* was confirmed as 4896 g/mol. In summary, the results demonstrated that the designed pH-sensitive polymer PBAE was successfully synthesized via the Michael-type polymerization.

**Figure 2.**
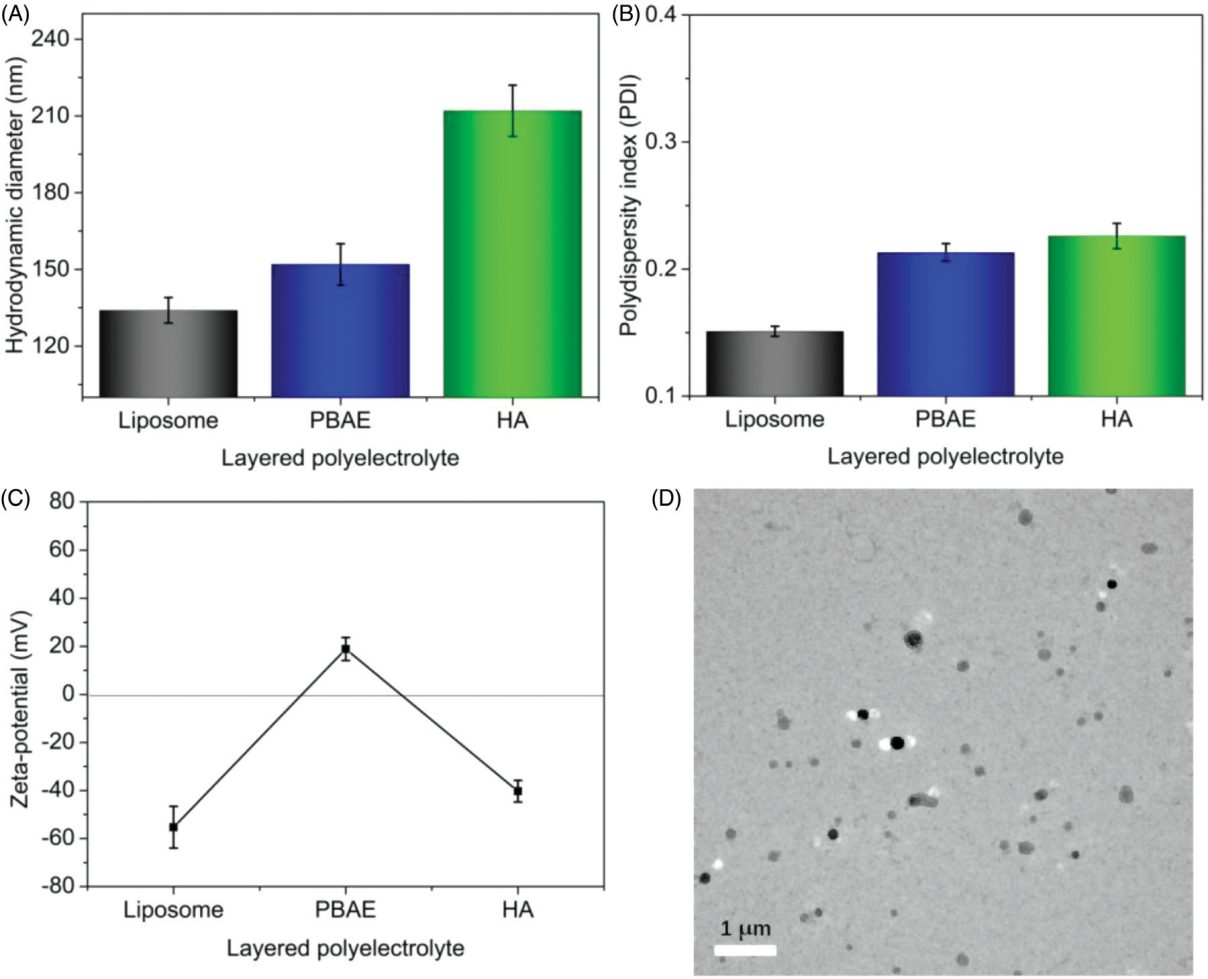
Preparation and characterization of DOX-loaded NPs. Hydrodynamic diameters (A), PDI (B), and charge reversal in zeta-potential (C) of DOX-loaded liposomes and DOX-loaded NPs. (D) TEM image of DOX-loaded NPs (scale bar: 1 μm).

Next, the multi-layered NPs and DOX-loaded NPs were prepared via LbL technique. First, DOX-loaded liposome was prepared through the pH gradient-dependent drug loading method, and then the PBAE layer and HA layer were coated via LbL through the polyelectronic interaction. The LbL polyelectrolyte coating process was recorded, and the results are shown in Figure 2. The hydrodynamic diameter of DOX-loaded liposomes was about 134 nm (Figure 2(A)). After the deposition of cationic PBAE layer, the particle size was increased to approximately 155 nm. After the deposition of anionic HA layer, the particle size of DOX-loaded NPs significantly increased to 212 nm. The increase of size indicated that the different functional layers were successfully coated on the surface of NPs. The PDI values of NPs after each deposition of functional layer were also recorded, as shown in Figure 2(B). The low PDI values (<0.30) exhibited the good uniformity of DOX-loaded NPs. Furthermore, the zeta-potential of NPs following each deposition was recorded, as shown in Figure 2(C). The zeta-potential of DOX-loaded liposomes without coating layer was about –55.3 mV, while it was significantly increased to +18.9 mV after coating of cationic PBAE layer. After deposition of anionic HA layer, the zeta-potential of NPs was decreased to negative (ca. –40.5 mV) again. As expected, a complete charge reversal following each layer deposition was observed (negative-positive-negative), indicating the successful PBAE/HA layers coating. Figure 2(D) presents the TEM image of the DOX-loaded NPs after incubation in PBS at pH 7.4 for 2 h at 37 °C. The DOX-loaded NPs displayed uniformly spherical in shape with good dispersity. The particle size (approximately 200 nm) was slightly smaller than that measured by DLS (212 nm), ascribing to the shrinkage of the NPs during TEM preparation. Collectively, multi-layered DOX-loaded NPs were successfully prepared using LbL technique with spherical morphology and good uniformity. Furthermore, the drug loading capacity of NPs was studied, and the results are shown in Table 1. EE was increased with the liposomes increasing, indicating the loaded DOX into liposomes were increased. But the LC was decreased with the liposomes increasing, possibly resulting from the largely increased weight of NPs after the deposition of PBAE and HA layers. At the mass ratio of 1:20 (DOX:liposome, m/m), the LC and EE were respectively 3.3% and 75.4%. However, the LC was increased to 4.7% and 5.6% while the EE were reduced to 72.5% and 56.1% at the mass ratio of 1:15 and 1:10. Therefore, the sample prepared at the mass ratio of DOX to carriers was 1:15 (4.7% for LC, 72.5% for EE) would be used in the follow study.

**Table 1.** LC and EE of DOX-loaded NPs at different mass ratios of drug and liposomes.

Liposomes (mg)	DOX (mg)	LC (%)	EE (%)
10	1	5.6	56.1
15	1	4.7	72.5
20	1	3.3	75.4

### 3.2. Confirmation of pH-sensitivity and stability

**Figure 3.**
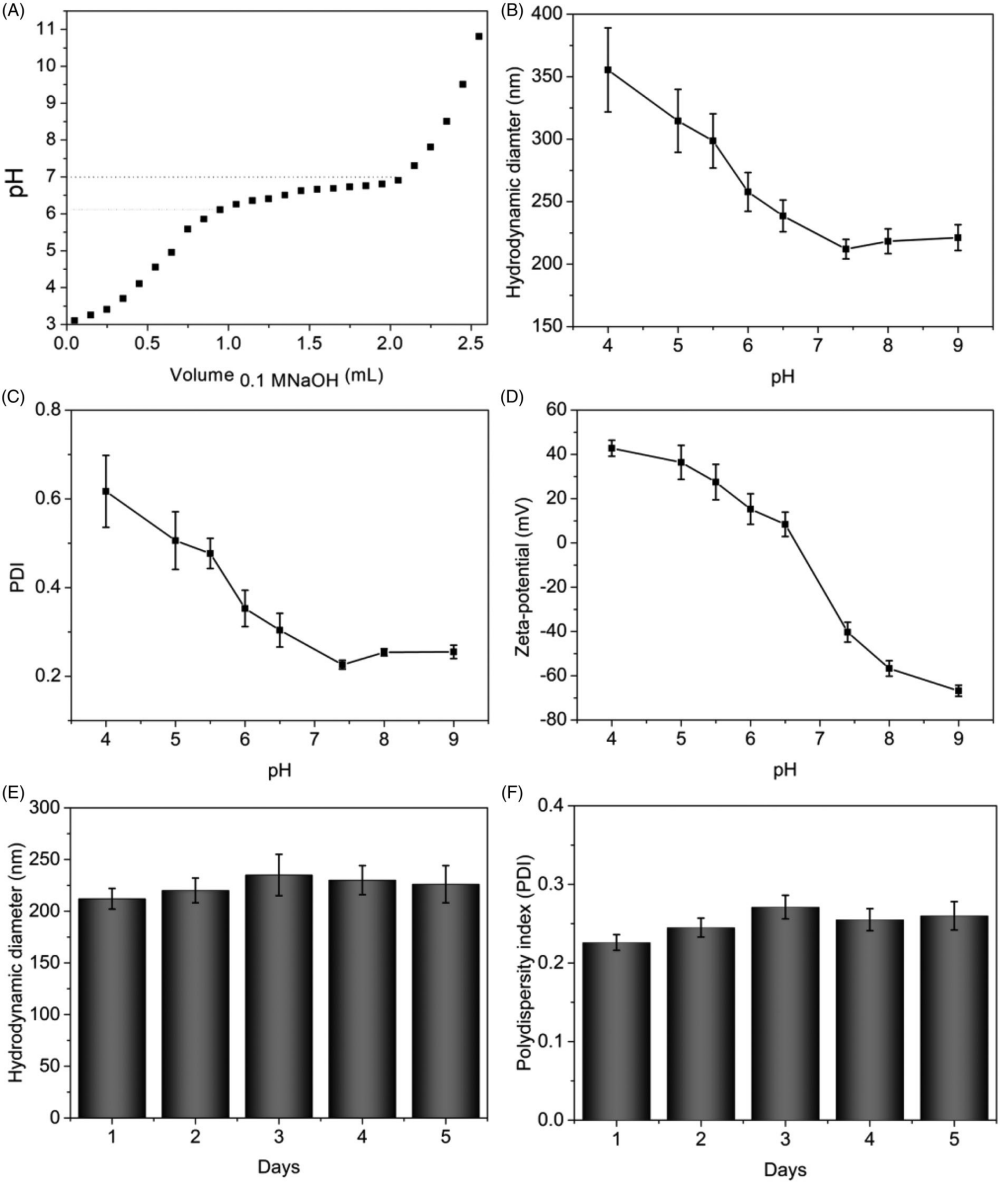
Confirmation of pH-sensitivity and stability of DOX-loaded NPs. (A) The potentiometric titration of the PBAE solutions dependent on the volume of NaOH solution. The particle size (B), PDI (C), and zeta-potential (D) of DOX-loaded NPs dependent on the different pH. Hydrodynamic diameters (E) and PDI (F) of DOX-loaded NPs after different incubation time in PBS with 20% FBS at 37 °C.

The tertiary amine residues of PBAE could be ionized in weakly acidic environment, resulting in pH-sensitivity of DOX-loaded NPs. To investigate this pH-sensitivity, we first determined the p*K*_b_ value of PBAE by an acid–base titration, as shown in Figure 3(A). At the beginning of NaOH addition, the pH value of solution was increased sharply. With the sequential addition of NaOH, the pH value of solution reached to a plateau in the range of 6.1–7.0, resulting from the protonation of tertiary amine residues in PBAE. The pH value of solution was increased obviously again with the addition of NaOH. The results indicated that the pH-sensitive range of PBAE was in the range of 6.1–7.0. As reported previously, the p*K*_b_ value of cationic polymer was defined as the solution pH at 50% neutralization of tertiary amine groups (Shen et al., 2009). Hence, the p*K*_b_ value of synthesized PBAE was calculated as 6.55. Next, we investigated the particles size, PDI and zeta-potential of DOX-loaded NPs at different conditions, as shown in Figure 3(B–D). With decrease of pH, the particle size of DOX-loaded NPs was dramatically increased, especially in weakly acidic environment. The reason could be that the tertiary amine residues in PBAE layer were protonated which leads to the solubility reversal of PBAE layer from hydrophobicity to hydrophilicity, resulting in swelling of DOX-loaded NPs. The PDI showed similar change trends due to the swollen and loose structure of DOX-loaded NPs with pH decreasing. As seen in Figure 3(D), the zeta-potential was changed from negative to positive and increased obviously when the pH decreased from base to acidic condition, resulting from the ionization of tertiary amine residues. To achieve high accumulative amount of NPs at site of tumor via EPR effect, the DOX-loaded NPs should have extended circulation time in body which indicated the NPs should have high serum stability. Thus, we evaluated the stability of DOX-loaded NPs after incubation in PBS at pH 7.4 with 20% FBS at 37 °C for five days. The hydrodynamic diameter and PDI were recorded in order to investigate the serum stability, as shown in Figure 3(E,F). After five days incubation, the hydrodynamic diameters of DOX-loaded NPs were in the range of 212–230 nm, and the PDI values were lower than 0.3. No obvious increase was observed. The negligible changes in particle size and PDI provided that the multi-layered DOX-loaded NPs prepared by LbL technique had high stability in serum solution which indicated that the NPs might be able to accumulate at the site of tumor for drug delivery. All the results demonstrated that the prepared DOX-loaded NPs exhibited pH-sensitivity with high serum stability which could be utilized for pH-triggered drug release.

### 3.3. *In vitro* pH-Triggered drug release profile

**Figure 4.**
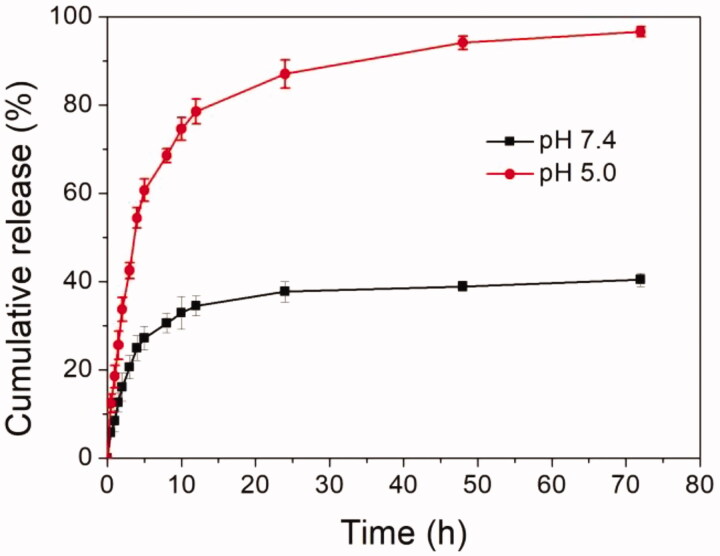
*In vitro* pH-triggered drug release profiles of multi-layered DOX-loaded NPs at pH 7.4 and 5.0.

Next, the DOX release profile from DOX-loaded NPs was investigated under normal physiological conditions (PBS, pH 7.4) and a slightly acidic environment (pH 5.0, intracellular tumor microenvironment), as shown in Figure 4. It could be obviously found that the drug release rate and cumulative release amount were significantly different as seen from the results. At the pH of 7.4, cumulative release of DOX was about 30% after 5 h and less than 40% after 72 h. The reasons could be that the pH-sensitive PBAE layer was not protonated and main part of DOX molecules was protected well in the liposome core and multi-layered NPs. However, when the pH decreased to pH 5.0, the DOX release rate was markedly accelerated compared to that at pH 7.4. The cumulative release of DOX was more than 60% after 5 h and about 98.8% after 72 h. The reasons might be that the tertiary amine residues in pH-sensitive PBAE layer were thoroughly protonated which made the hydrophilic PBAE middle layer, leading to unprotected DOX-loaded liposome. The DOX molecules were rapidly released from the core. Moreover, the low pH of external medium facilitated the release of DOX from core of NPs. In summary, the DOX-loaded NPs could protect DOX molecules well at pH 7.4 and release the cargos when the pH was decreased to weakly acidic condition, indicating the DOX release from NPs was pH-triggered. These findings suggested that the DOX could be controlled release from multi-layered pH-sensitive DOX-loaded NPs by responding to the weakly acidic cue.

### 3.4. Cellular uptake

**Figure 5.**
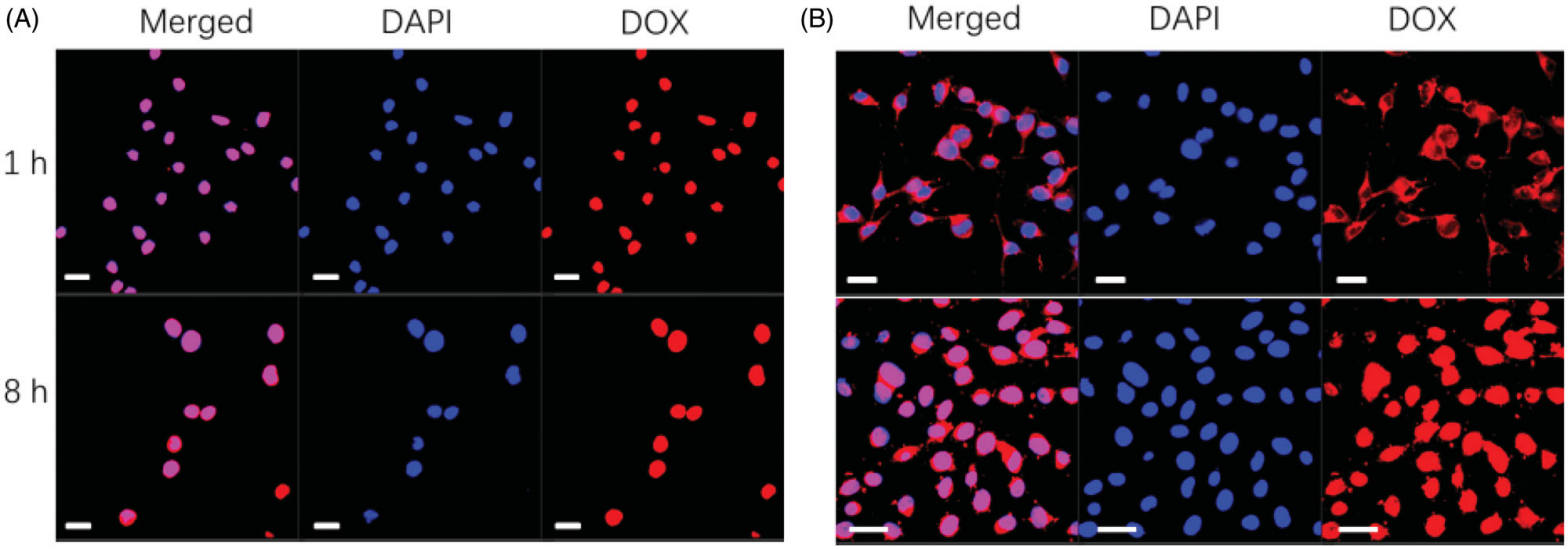
Cellular uptake of free DOX and DOX-loaded NPs. Confocal microscopy image of cells incubated with free DOX (A) and LbL DOX-loaded NPs (B) for different time intervals (upper: 1 h, bottom: 8 h, scale bars are 20 μm).

In order to efficiently induce the apoptosis of tumor cells, the anticancer drug DOX should be targeted delivery into intracellular and bind with tumor cell nucleus. Herein, the intracellular localization of free DOX and DOX-loaded NPs against A549 cells at different time was studied by CLSM, as shown in Figure 5. When the free DOX was incubated with A549 cells for 1 h (Figure 5(A), upper), DOX molecules were mainly distributed in the cell nucleus region which indicated that the DOX molecules could induce the death of A549 cells effectively. After incubation for 8 h, similar phenomenon was observed for free DOX (Figure 5(A), bottom). By contrast, at 1 h post-incubation with DOX-loaded NPs (Figure 5(B), upper), the DOX molecules were mainly distributed in the cytoplasm because most DOX molecules were kept in the core of NPs (only about 20% of DOX molecules were release, Figure 4). However, after incubation for 8 h (Figure 5(B), bottom), strong fluorescence intensity was obviously detected in whole A549 cells including cell nucleus region, suggesting that mainly DOX molecules were fastened to the nucleus, resulting from more than 70% of DOX molecules were released (Figure 4). DOX-loaded NPs were able to target to the A549 cells and bind on the surface of cells due to the specific interaction between HA shell and CD44 receptor, facilitating the cellular uptake of NPs by tumor cells that resulted in similar distribution of DOX molecules compared to free DOX at 8 h post-incubation. In summary, these findings demonstrated that the DOX-loaded NPs could be internalized effectively by A549 cells and deliver DOX molecules to cell nucleus, indicating that the multi-layered pH-sensitive NPs might be potential targeted anticancer drug delivery carrier with controlled release profile.

### 3.5. Cytotoxicity assay

**Figure 6.**
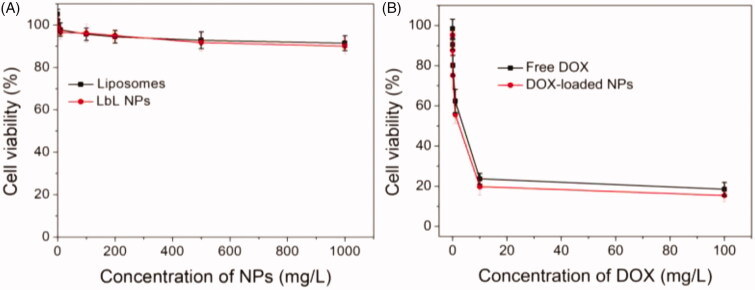
*In vitro* cytotoxicity of liposome and NPs (A), free DOX and DOX-loaded NPs (B) at different concentrations against A549 cells for 24 h.

The promising drug delivery carriers for cancer therapy should be low cytotoxic or nontoxic, while the cytotoxicity of drug-loaded system based on the carriers against tumor cells should be high. Next, we evaluated the cytotoxicity of the liposomes, NPs, free DOX, and DOX-loaded NPs against A549 cells using MTT assay (Figure 6). As shown in Figure 6(A), although the cytotoxicity of liposome and NPs for A549 cells was slightly enhanced with the increase of concentration, the cell viability was still higher than 90% even at the highest concentration (1000 mg/L), indicating that both of liposome and NPs showed very low and negligible cytotoxicity. The cytotoxic effect of free DOX and DOX-loaded NPs against A549 cells for 24 h is shown in Figure 6(B). The results exhibited that the cytotoxicity of DOX-loaded NPs was similar to that of free DOX in the treatment of 24 h, resulting from that the DOX could be released from the NPs (Figure 4) and the released DOX molecules could work as free ones (Figure 5). In summary, the NPs coated with pH-sensitive PBAE layer and targeting HA layer had negligible cytotoxicity, and the DOX-loaded NPs showed high growth inhibition effect against A549 in comparison to free DOX.

### 3.6. Antitumor evaluation *in vivo*

**Figure 7.**
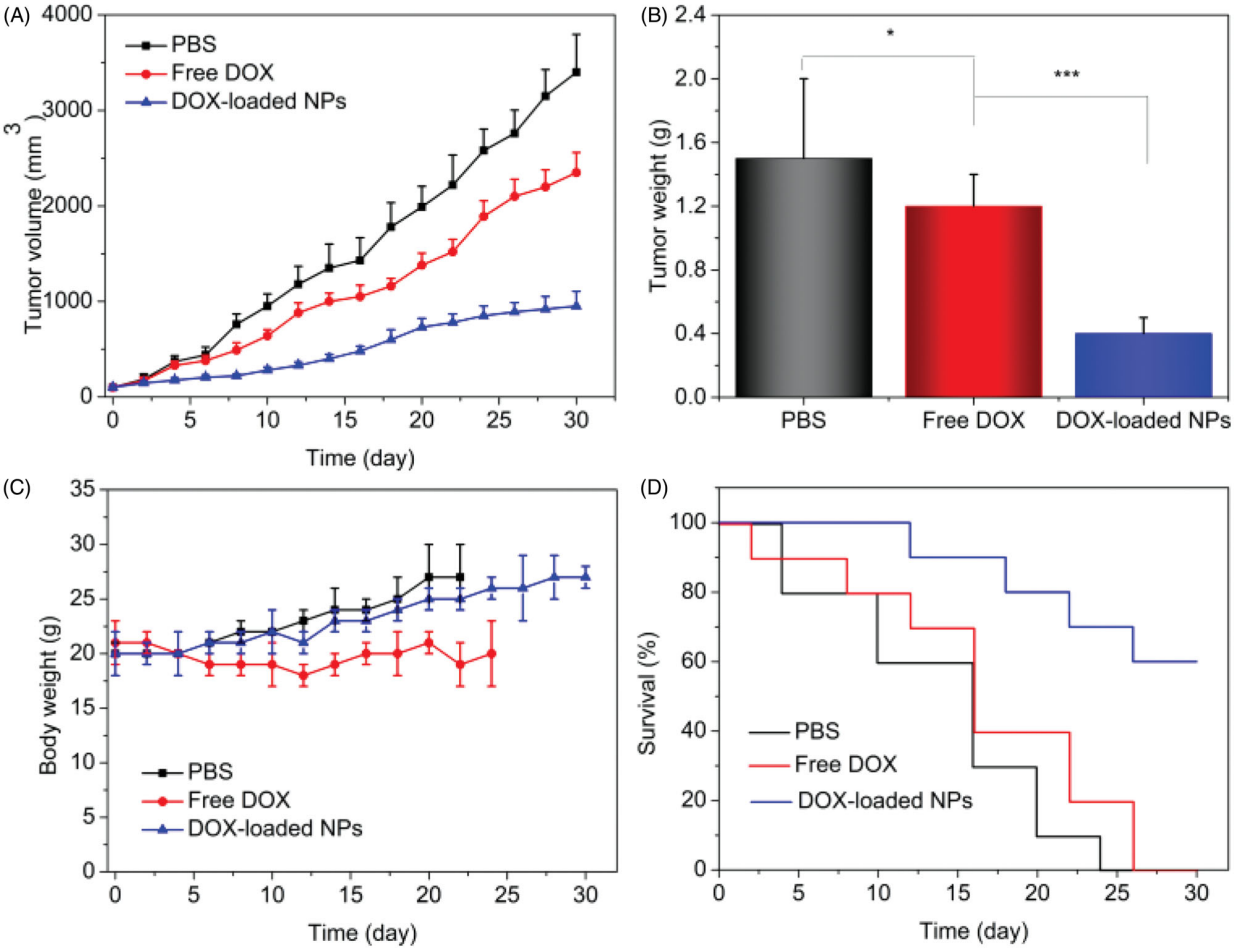
Therapeutic efficacy of A549 tumor-bearing mice. (A) Tumor volume growth curves of different groups of mice after various treatments; (B) average weights of tumors collected from the mice at the end of therapy; (C) the weight of A549 tumor-bearing mice (*n* = 10, mean ± SD). *p< .05, ***p< .001. (D) Survival rates of mice after treatment. Statistical analysis was done using Kaplan–Meier’s method (*n* = 10).

Since the pH-triggered drug release profile and intracellular uptake of DOX-loaded NPs have been confirmed, the *in vivo* anticancer efficiency was investigated here. When the tumor size reached approximately 100 mm^3^, the tumor-bearing mice were randomly divided into three groups: PBS i.v. injection, free DOX (4 mg/kg) i.v. injection, and DOX-loaded NPs (equal to 4 mg/kg of DOX) i.v. injection. The tumor sizes of mice with different treatments were measured, as shown in Figure 7(A). The tumor volume of PBS-treatment group grew sharply. The growth of tumor was slightly inhibited after administration of free DOX. By contrast, the tumor size of DOX-loaded NPs-treatment group was significantly inhibited in comparison to PBS and free DOX treatment. After 30 days of treatment, the mean weights of tumor in each group are shown in Figure 7(B). The mean tumor weight of free DOX-treatment group (1.2 g) was slightly less than that of group treated with PBS (1.5 g). However, the mean tumor weight of group treated with DOX-loaded NPs was only about 0.4 g, which was much less than those of PBS and free DOX-treatment groups. These findings revealed that the DOX-loaded NPs possessed the most effective tumor inhibition effect, resulting in improved therapeutic efficacy comparing to PBS and free DOX. In order to evaluate the safety of nanotherapeutics, we have measured the cytotoxicity of blank NPs and DOX-loaded NPs *in vitro*. Here, we further confirmed the safety of prepared DOX-loaded NPs. The body weights of three groups with different treatments were monitored, as shown in Figure 7(C). The mice treated with free DOX displayed obvious weight loss comparing with those treated with PBS and DOX-loaded NPs, resulting from severe side-effect caused by off-targeting effect. On the contrary, the mice treated with DOX-loaded NPs exhibited similar growth trend in comparison to those treated with PBS, indicating the low unwanted toxicities. In addition, the weight of the heart or liver of free DOX-treated group was decreased in comparison to others, indicating the toxicity of the free DOX (Figure S4). Furthermore, the results of blood biochemistry analysis showed that some key factors including heart function marker (CK), hepatic function markers (ALT, AST), and renal function markers (CREA, BUN) of mice treated with free DOX were markedly different from those of normal control, while DOX-loaded NPs-treated group showed no distinction, as shown in Figure S5. Summarily, the DOX-loaded NPs did not exhibit obvious toxicity compared to free DOX treatment, suggesting the safe application of prepared DOX-loaded NPs system. Finally, the *in vivo* survival rates of PBS, free DOX, and DOX-loaded NPs are recorded as shown in Figure 7(D). For the treatment of PBS, all the tumor-bearing mice were dead at 24 days. The survival of free DOX-treatment group was 20% at 24 days and died at 26 days. In contrast, the survival of group treated with DOX-loaded NPs was still 100% at 12 days, and decreased to 70% at 24 days. Even after 30 days, the survival was still 60%. These results suggested that the DOX-loaded NPs had much higher anti-tumor efficacy compared to free DOX. In summary, the prepared NPs could improve the therapeutic efficacy with reduced side-effect and enhance the survival rate of the tumor-bearing mice in comparison to free DOX.

## 4. Conclusions

In the present work, the cationic pH-sensitive polymer PBAE was first synthesized via the Michael-type polymerization. The anticancer drug DOX was then efficiently loaded in the liposomes with high LC and EE through the pH gradient. Next, the multi-layered pH-sensitive DOX-loaded NPs were prepared by LbL technique and the process was confirmed by measurement the particle size and zeta-potential following each layer deposition. The hydrodynamic diameter was increased as pH-sensitive PBAE layer and targeting HA layer was coated in sequence with good uniformity and specific spherical morphology. What is more, a complete charge reversal following each layer deposition (negative-positive-negative) was detected to further validate of the coating of each functional layer. The negative surface charge and high serum stability suggested the DOX-loaded NPs could have prolonged circulation time and enhanced accumulation at site of tumor. The p*K*_b_ value of polymer PBAE was about 6.5, and the particle size and zeta-potential of DOX-loaded NPs was obviously increased with the decrease of pH that was caused by the protonation of tertiary amine residues in PBAE at weakly acidic condition, demonstrating the pH-sensitivity of multi-layered DOX-loaded NPs. As expected, the DOX molecules release profile from multi-layered NPs was dependent on pH. The release rate at low pH was significantly accelerated in comparison to that at base or normal pH, indicating the potential for drug controlled release. Next, the DOX-loaded NPs were provided to effectively deliver cargos to A549 cells via CLSM imaging. After incubation for 8 h, the DOX molecules were deposited at the cell nucleus by multi-layered NPs comparing with the free DOX. The *in vitro* cytotoxicity of carriers and DOX-loaded NPs against A549 was studied, and the results exhibited that the carriers showed very low toxicity and DOX-loaded NPs had similar cytotoxic effect comparing with free DOX. The *in vivo* therapeutic experiment demonstrated that the DOX-loaded NPs showed the best anticancer efficacy with reduced side-effect in comparison to free DOX and PBS because of active targeting effect of HA and pH-triggered drug release behavior. These results suggested that the multi-layered pH-responsive NPs might be promising and efficient drug delivery carrier for cancer chemotherapy, and the LbL technique could be a useful method to prepare platform for drug delivery and controlled release.

In this study, the multi-layered liposome–polymer hybrid NPs with pH-sensitivity and targeting effect were designed and prepared successfully via LbL polyelectrolyte coating processes for drug delivery and controlled release. Anticancer drug DOX was effectively loaded, resulting in DOX-loaded NPs. The DOX-loaded NPs were efficiently internalized by A549 cells, and the DOX molecules were controlled release from multi-layered NPs triggered by low pH and deposited at cell nucleus to induce the death of tumor cells. In summary, the multi-layered NPs could be potential delivery carriers for cancer chemotherapy. Furthermore, the LbL method could be efficient way to prepare functional liposome–polymer hybrid platform for drug delivery and controlled release.

## Supplementary Material

Supplemental Material
