# Supplementary material for: Layer-by-layer pH-sensitive nanoparticles for drug delivery and controlled release with improved therapeutic efficacy in vivo
Source: Drug Deliv. 2020 Jan 10;27(1):180–90. doi: 10.1080/10717544.2019.1709922 (PMC7008239; doi:10.1080/10717544.2019.1709922)
Supplement: Supplemental Material [file IDRD_A_1709922_SM5055.docx]

**Supporting Information**

**Layer-by-layer pH-sensitive nanoparticles for drug delivery and controlled release with improved therapeutic efficacy in vivo**

Wanfu Men, Peiyao Zhu, Siyuan Dong, Wenke Liu, Kun Zhou, Yu Bai, Xiangli Liu, Shulei Gong, Shuguang Zhang*

Department of Thoracic Surgery, The First Affiliated Hospital of China Medical University, Shenyang 110001, People's Republic China

*Corresponding Author:

Shuguang Zhang, Department of Thoracic Surgery, The First Affiliated Hospital of China Medical University, No. 155 North Nanjing Street, Heping District, Shenyang 110001, Liaoning Province, P.R. China. E-mail: shgzhang@cmu.edu.cn.

The authors declare no competing financial interest.


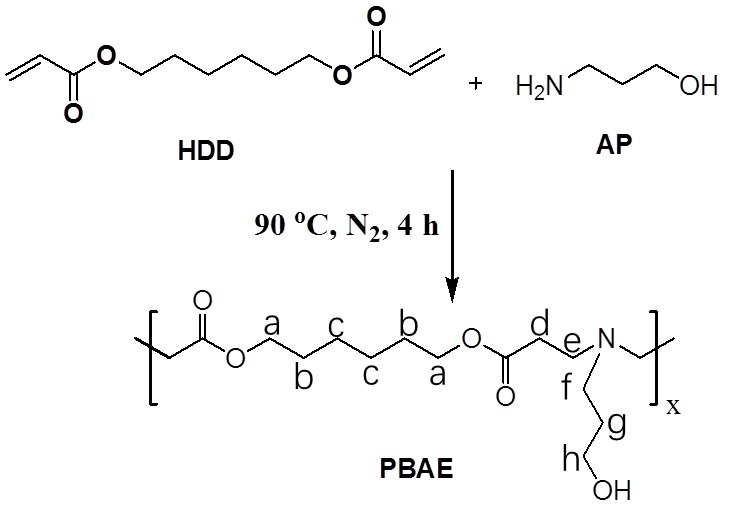


**Figure S1.** Synthetic route of pH-sensitive polymer poly(*ß*-amino ester) (PBAE).


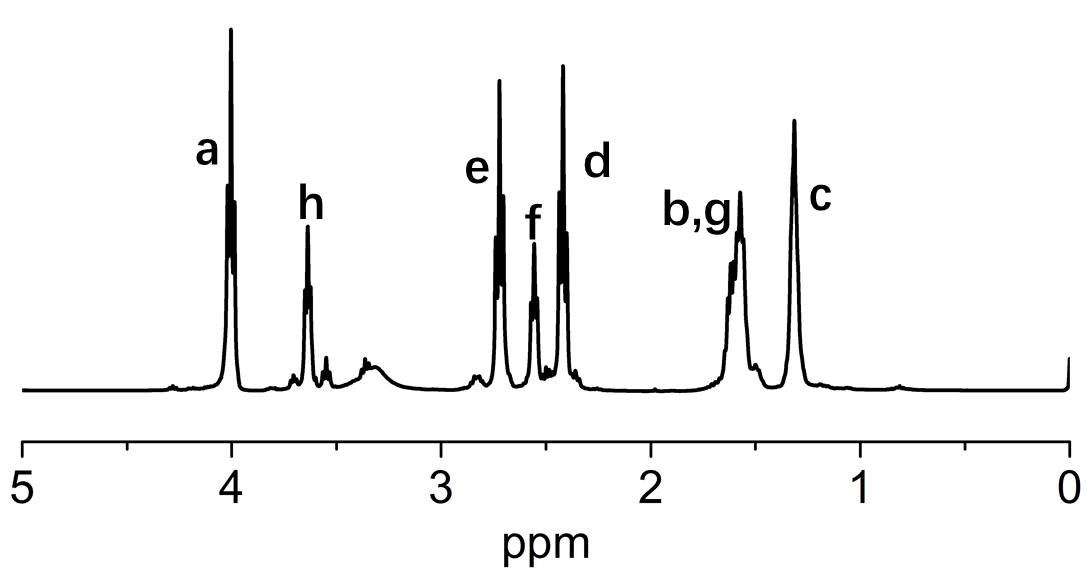


**Figure S2**. ^1^H-NMR spectrum of poly(*ß*-amino ester) (PBAE) in CDCl_3_-*d*.


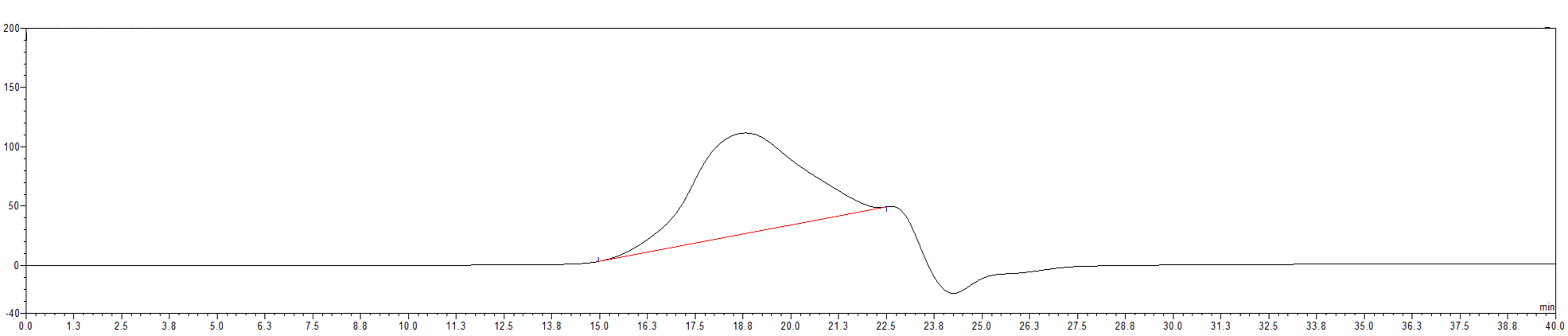


**Figure S3.** GPC trace of poly(*ß*-amino ester) (PBAE) in THF.


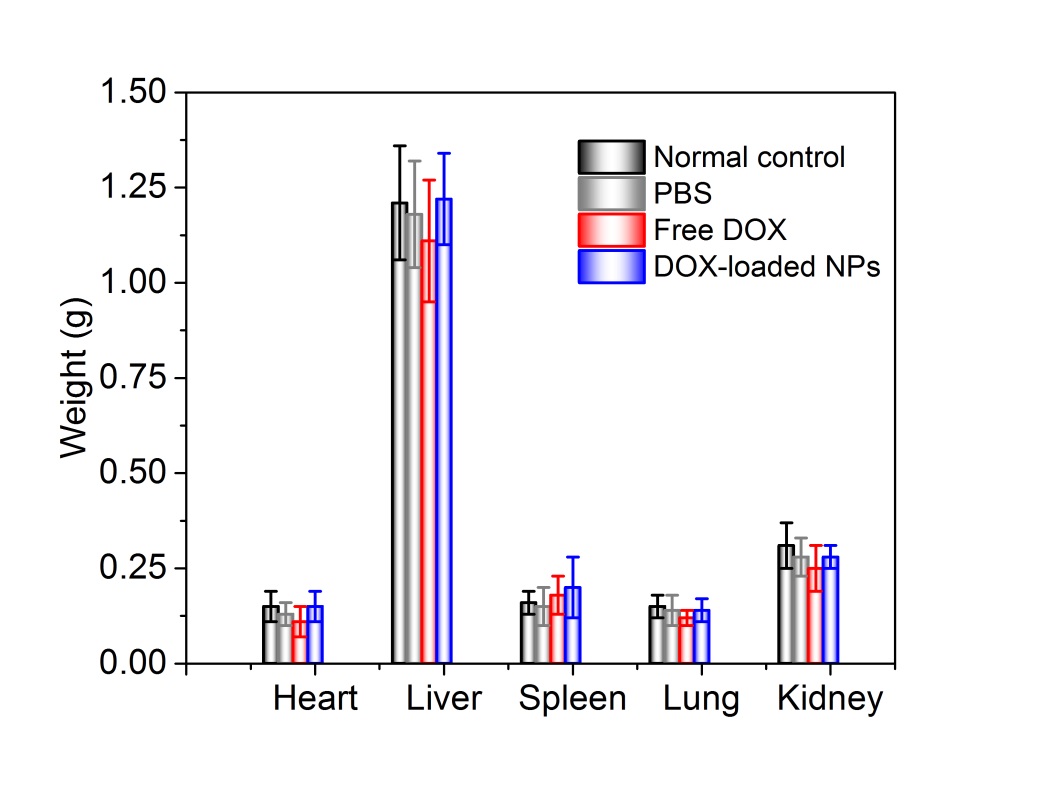


**Figure S4.** The weight of major organs of mice treated with different solutions.

**
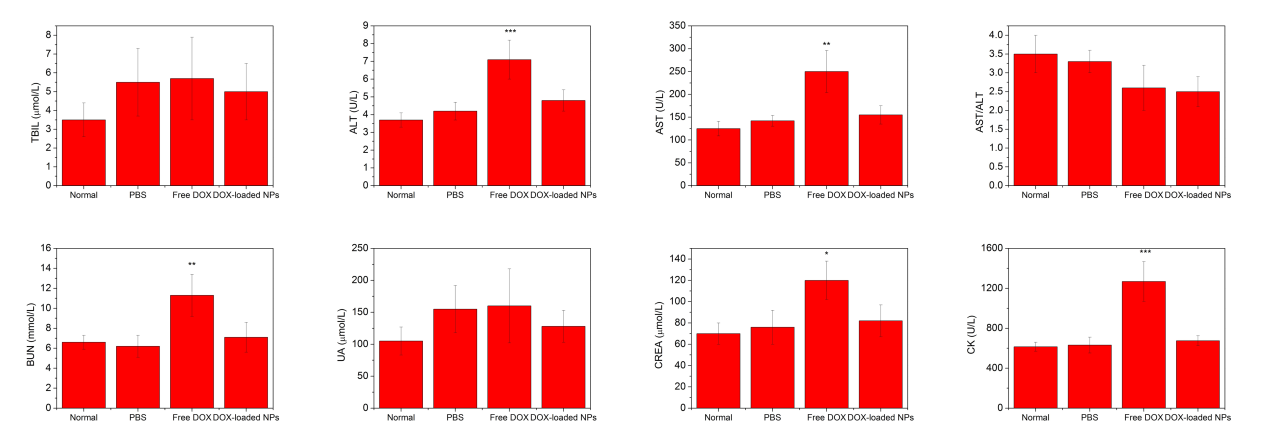
**

**Figure S5.** Blood biochemistry analysis of the mice treated with PBS, free DOX and DOX-loaded NPs, respectively. The results show mean and standard deviation of total bilirubin (TBIL), aminotransferase (ALT), aminotransferase (AST), blood urea nitrogen (BUN), uric acid (UA), creatinine (CREA), creatine kinase (CK). *P* values: * *p*< 0.05, ***p*< 0.01, ****p* < 0.001.
